# Obstetrics

**Published:** 1879-01

**Authors:** 


### OBSTETRICS.

OBSTETRICAL ANÆSTHESIA. E. Hervieux. (*L'Union Médicale, Aug.* 27, 1878.)

The author is a partisan of obstetrical anæsthesia, and considers it as a veritable conquest of modern medicine. He protests against the opposition it has met with in France, but under conditions: 1st, that the indications and contraindications be clearly stated, and 2d, that the accoucheur always superintend personally the administration of the chloroform. Certain colleagues have presented obstetrical anæsthesia as practiced now-a-days, as completely inoffensive. This is an exaggeration and may cause the most dangerous consequences, of which the least would be to compromise the cause we would serve.

Undoubtedly there is no comparison to establish between surgical and obstetrical anæsthesia. The latter, as Campbell says, is only an analgesia. It does not abolish the intellectual and sensorial faculties; it does not take away the consciousness of self. It requires to obtain it (and this is capital) but very feeble doses of chloroform. It may be kept up for hours without difficulty, and without giving rise to coma or the stertor of surgical anæsthesia. The danger is then reduced to the smallest proportions, but not entirely removed. These are the proofs:

1st. There are subjects in whom the sensibility to the action of chloroform is excessive, and who only need a few inspirations of the anæsthetic agent to lose sense of feeling and consciousness. The author cites his own case in which the inhalation of chloroform for the extraction of a tooth, for about fifteen seconds plunged him into a profound sleep, and absolute insensibility. Cases of this kind are common with children, and not very rare in young women. Suppose an accoucheur has a case of this kind, and, encouraged by former successes, departs from the rules of prudence and gives large doses after the example of Simpson. Is it not evident that he may be surprised by comatose symptoms capable of endangering the life of the patient? The obstetrical effort may indeed, as Campbell says, bring the patient out of the coma, but this effort may be lacking as well as the uterine contraction which follows it, and the danger then cannot be contested.

2d. In opposition to the exaggerated sensibility of certain women, there are organisms refractory to this action. I have met with some patients with an invincible resistence to the anæsthetic power of chloroform—invincible, at least, as regards the limits which I laid down for myself. It has seemed to me as if women, familiarized for a long time with the use of ether, or those who, under pretext of a need of tonics, have taken daily a considerable quantity of pure wine, are overcome by the anæsthetic agent with great difficulty. The author cites the case of a young woman to whom he gave about 100 grams of chloroform, obtaining but very incomplete anæsthesia. There is in this natural or acquired resistence to the action of chloroform, a danger which should be understood, for if one seeks only analgesia during the labor, he would only succeed by such an accumulation of doses that, at a given moment, he might no longer be master of the situation.

3d. I have encountered certain women who, after having solicited chloroform, had an excessive repugnance for absorption of its vapor. If one does not proceed like Simpson with large doses so as to plunge the patient rapidly into the anæsthetic state, it may happen that the patient will not take the chloroform, having been painfully impressed with the first inhalations, and not having had time to feel its benefits.

4th. Chloroform retards the labor. M. Campbell has recognized this fact, and observations made recently at the Maternité by M. Pinard confirm it. The intervals which separated each contraction were noted with care during one or several hours, then chloroform was given in moderate doses, and under its influence the internal instead of diminishing progressively, as occurs in a natural labor, gradually increased. There was one case, even, in which there was complete cessation of pains, and use of forceps necessitated. Now suppose the bag of water to be prematurely broken, it is not simply a momentary arrest of uterine contractions that we have, but the death of the fœtus by asphyxia is threatened.

Is there not still another danger to be noted? at the same time recognizing with Campbell: 1st. That the lengthening of the intervals and the lessening of their duration and intensity by chloroform are especially observed in cases of exceptionally rapid and painful dilatation. 2d. That the labor, momentarily retarded, often resumes its normal rhythm of contractions and efforts which cause toward the end an accelerated movement, so as to compensate for the primitive retardation. From this we see that the cases of retarded labor observed by Pinard are in no wise contradictory to those of acceleration noted by Dumontpallier, since the retardation determined by chloroform may have taken place during the period of dilatation and the accelerated movement in the expulsory stage.

5th. There are women originally disposed to syncope, in whom the state of gestation, even the most normal, aggravates in a singular manner this disposition. Suppose that this fact be unknown to the accoucheur, is it not evident that chloroform would be dangerous ? The disposition to syncope might be complicated with extreme susceptibility to chloroform, and this double idiosyncrasy might act simultaneously to compromise the essential functions from the very first inhalations.

6th. Beside the syncopal tendency must be mentioned, as so many dangers suspended over the head of the patient, all the diseases of the heart and respiratory tract which predispose to asphyxia, hypertrophy of the heart, valvular narrowings, tuberculosis, emphysema, pleuritic effusions, etc. It is true, Championnière does not believe that most of these affections contra-indicate the use of chloroform. But is this a reason why we should regard chloroform as innocuous in these diverse pathological states? We may brave a real peril ten, twenty times, but a day comes when by the neglect of a simple precaution or detail, we succumb. I might mention all the diseases of the nervous centers which give rise to passive hyperæmias, such as cerebral hæmorrhages, softening of the brain, etc. Also certain physiological or morbid states, such as a twin pregnancy, hydropsy of the amnion, and in general all the organopathic conditions which distend beyond measure the abdomen, crowding back the diaphragm and with it the heart and lungs, and causing syncope, asphyxia or visceral congestions.

7th. Finally, there is a danger almost as much to be feared as most of those just mentioned, that which arises from a deficient obstetrical education. To administer the chloroform with measure, to keep, by means of suitable doses methodically given, the patient in a state of analgesia, which does not take away from her the exercise of her intellectual functions, and her consciousness, is very well. But if, by reason of faulty diagnosis, which is perhaps most commonly the case, the accoucheur overlooks a grave cause of distocia, deformed pelvis, presentation of the trunk, locking of the head by fault of rotation, etc., or any other circumstance requiring an operation, what will happen? The prolongation of the anæsthesia will bring on a situation as perilous for the mother as for the child, perilous because one waits instead of acting; perilous because the resistance of the obstacle annihilates the power of the obstetrical effort; perilous because this effort no longer produced, no longer exercises its disanæsthetic action upon the organism; perilous finally, because every labor which does not terminate, exhausts the forces of the mother and compromises the existence of the fœtus.

The author would not, therefore, entrust the administration of chloroform to internes and nurses in the absence of the physician, nor does he consider it advisable to allow midwives to give anæsthetics except under the advice and direction of the physician. Having reviewed the dangers and inconveniences of obstetrical anæsthesia, the author speaks of the advantages of this restricted anæsthesia.

1st. *Chloroform lessens the pains of uterine contraction.*— All the efforts of adversaries cannot prevail against this fact, slight in appearance, but undeniable, and great in practical consequences. They say it is not true anæsthesia. What matters it if the analgesia obtained is sufficient to make the patient bear patiently the pains so dreaded and so full of anguish? They say, also, that in this restricted anæsthesia the patient still suffers. True, but there are degrees in suffering. If, instead of suffering as ten I only suffer as one, should I not esteem as an immense benefit the agent that can reduce the pain in such a degree? The lessening of the pain by chloroform is not subject to an inflexible law; it may vary with individual susceptibilities, the doses being equal. The fact is beyond dispute.

2d. *Chloroform procures to the woman in labor a perfect calm during the interval of uterine contractions.*—M. Campbell describes the interval between contractions in this semi-anæsthesia as *a grand silence.* M. Lucas Championnère, Pinard and the author, have all observed that the patient remains perfectly calm during the interval.

3d. *Semi-anæsthesia helps the patient to pass easily through the painful period of dilatation.*—The first stage of this period is usually easily borne, the pains being short and slight, and the intervals long and painless. But in the second stage the pains are sharper and more frequent, and the intervals marked by increasing agitation; the woman suffers all the time, weeps, despairs, perceives no progress, and declares she can never stand it. Now, chloroform in small doses calms this agitation, dissipates fear and evil presentments, and allows the nervous system, shaken by the shocks of pain, time to recover its physiological equilibrium. M. Danyau is one of the first to make the possible application of anæsthesia to the period of dilatation. He says: "I think we may have recourse to simply attenuating doses of chloroform in cases of very slow and painful dilatation, even if we renounce it later, at the beginning of the expulsory stage, in general, much less painful, and moreover nearly always so valiantly supported by women."

4th. *Chloroform renders tolerable the great pains of the expulsory stage.*—While many women do support this stage well, yet, in all cases where the expulsory stage is long and painful, and especially in primiparæ, the author regards chloroform as a great boon, and if we did not have chloroform it should be discovered.

5th. *The certitude of being given chloroform frees the primiparæ from the terrors of labor. It gives to multiparæ, who have already experienced the benefits of anæsthesia a salutary confidence.*—True, most primiparæ do not dread parturition. They know they must suffer, but have a vague idea of the pains that await them, feeling, perhaps, that where the mother has passed the daughter may also pass. But there are others who regard it with terror, believing they shall never be able to live through it. Give such persons the assurance that chloroform will spare them these pains, and their confidence is established and all their gloomy thoughts will vanish like a bad dream.

The multipara also, whether previously anæsthetized or not, on the promise of being given chloroform, will pass through the diverse stages of gestation full of confidence and security. It is well known what a happy influence the absence of inquietude and of all preoccupation has upon pregnancy.

6th. *Chloroform is one of the greatest aids in nervous or hysterical women, with exaggerated sensibility, whom the pains of childbirth throw into extreme agitation.*—The author gives the following case to illustrate the good effects of chloroform in a highly nervous woman: An extremely nervous young woman, pregnant for the third time, and at term, was taken during the period of dilatation with disordered movements which her husband, the nurse and myself could with the greatest difficulty repress. When left to herself, she sat up and lay down, turned and rolled about constantly, tossed about, threw her arms right and left, kicked, and in a word, gave herself up to the most violent gymnastics, ever crying, weeping, gesticulating. To put an end to these fantastic exercises he resorted to chloroform which produced complete quiet. Expulsion soon took place, during a relative calm, the uterine contraction being valiantly aided by the muscular efforts of the patient. He employed 80 grams of chloroform and there was neither sleep, nor period of excitation, nor abolition of consciousness.

7th. *Chloroform will also be very useful in all cases of delay or suspension of labor, whether from nervous exhaustion, local pains, neuralgic or otherwise, rigidity or spasmodic retraction of the neck, or from the effects of partial or irregular contractions of the womb.*—In addition to his own experience, the author refers to that of Dumontpallier, Cazeaux, Montgomery, Meigs, to support the truth of this statement.

It results from the preceding declarations that chloroform in obstetrics has numerous indications, and that the number of its advantages is far greater than the dangers. The more we advance in the discussion of this question, the more we enlarge the circle of its indications, and the better they are pointed out. It would be imprudent, almost unskillful, to use chloroform in all cases without distinction. The contra-indication might emanate from not only certain abnormal conditions, but also from physiological circumstances, such as rapid and painless labor.

